# The Role of Kynurenine Pathway and NAD^+^ Metabolism in Myalgic Encephalomyelitis/Chronic Fatigue Syndrome

**DOI:** 10.14336/AD.2021.0824

**Published:** 2022-06-01

**Authors:** Mona Dehhaghi, Hamed Kazemi Shariat Panahi, Bahar Kavyani, Benjamin Heng, Vanessa Tan, Nady Braidy, Gilles J. Guillemin

**Affiliations:** ^1^Neuroinflammation Group, Faculty of Medicine and Health Sciences, Macquarie University, NSW, Australia.; ^2^PANDIS.org, Australia.; ^3^Centre for Healthy Brain Ageing, School of Psychiatry, University of New South Wales, Sydney, Australia.

**Keywords:** Kynurenine pathway, tryptophan, NAD^+^, myalgic encephalomyelitis/chronic fatigue syndrome, gut microbiota

## Abstract

Myalgic encephalomyelitis/chronic fatigue syndrome (ME/CFS) is a serious, complex, and highly debilitating long-term illness. People with ME/CFS are typically unable to carry out their routine activities. Key hallmarks of the disease are neurological and gastrointestinal impairments accompanied by pervasive malaise that is exacerbated after physical and/or mental activity. Currently, there is no validated cure of biomarker signature for this illness. Impaired tryptophan (TRYP) metabolism is thought to play significant role in the pathobiology of ME/CFS. TRYP is an important precursor for serotonin and the essential pyridine nucleotide nicotinamide adenine dinucleotide (NAD^+^). TRYP has been associated with the development of some parts of the brain responsible for behavioural functions. The main catabolic route for TRYP is the kynurenine pathway (KP). The KP produces NAD^+^ and several neuroactive metabolites with neuroprotective (*i.e.*, kynurenic acid (KYNA)) and neurotoxic (*i.e.*, quinolinic acid (QUIN)) activities. Hyperactivation of the KP, whether compensatory or a driving mechanism of degeneration can limit the availability of NAD^+^ and exacerbate the symptoms of ME/CFS. This review discusses the potential association of altered KP metabolism in ME/CFS. The review also evaluates the role of the patient’s gut microbiota on TRYP availability and KP activation. We propose that strategies aimed at raising the levels of NAD^+^ (*e.g.*, using nicotinamide mononucleotide and nicotinamide riboside) may be a promising intervention to overcome symptoms of fatigue and to improve the quality of life in patients with ME/CFS. Future clinical trials should further assess the potential benefits of NAD^+^ supplements for reducing some of the clinical features of ME/CFS.

## 1.Introduction

**Figure 1.**
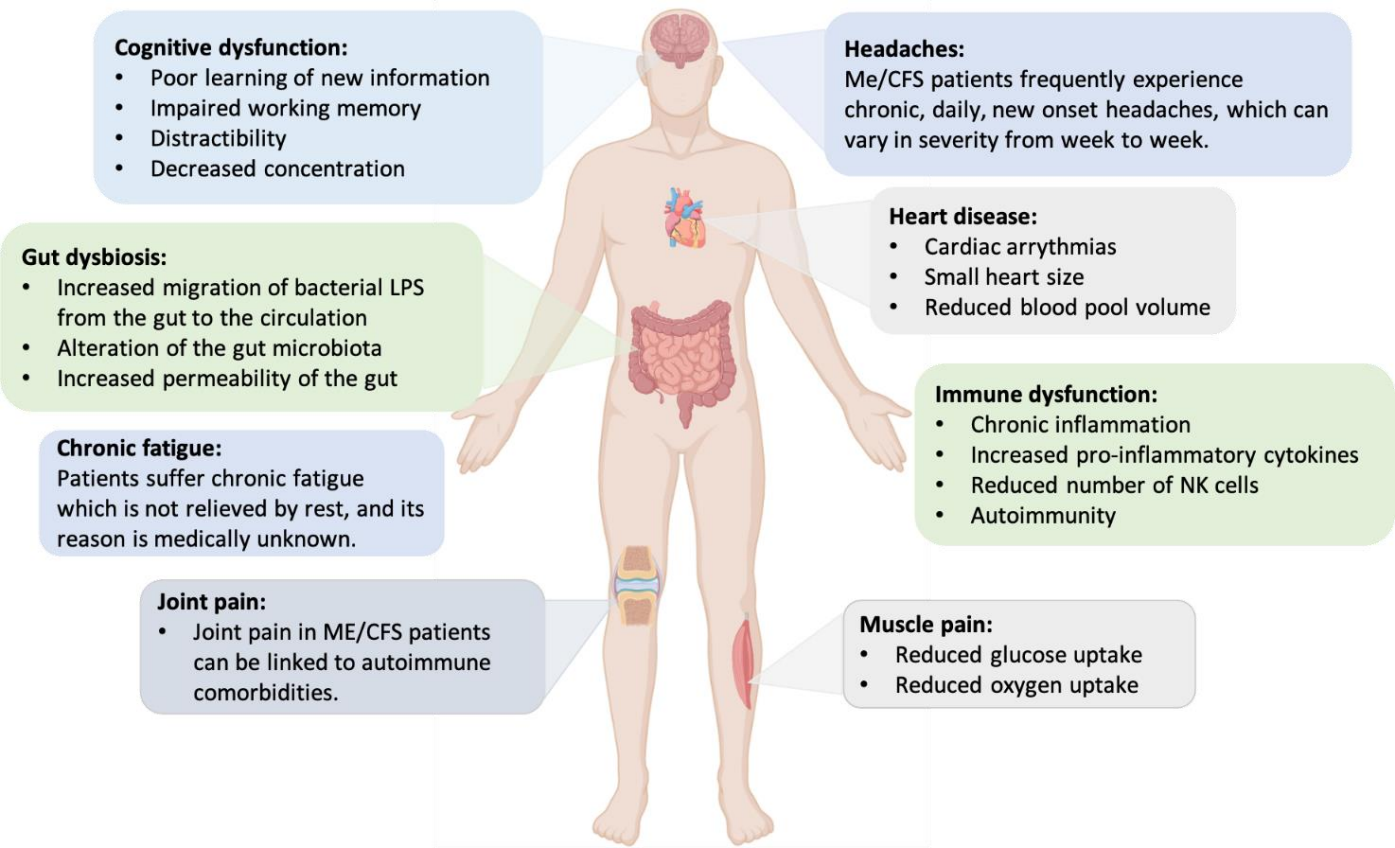
Common symptoms and significant features of ME/CFS patients.

Myalgic encephalomyelitis/chronic fatigue syndrome (ME/CFS) is a complex multisystem, long-term, disabling disorder. The key hallmark of ME/CFS is debilitating fatigue that is present for at least 6 months and is not ameliorated by rest. [1]. According to the Institute of Medicine (IOM) report, 83,000 to 2.5 million individuals suffer from ME/CFS in the United States with an estimated annual economic burden of 17-24 billion USD dollars [2]. It is estimated that the prevalence of ME/CFS to be 0.4-1% of the Australians, implying up to 250,000 people affected [3]. This disease is characterized by complicated symptoms associated with impairment in cognition, immune system, autonomous functions, and the endocrine system (Fig. 1). Individuals with ME/CFS suffer overwhelming fatigue, dizziness, and sleep disturbances [4]. The illness can affect all races and ages with women at three to four times higher risk than men [5-7]. Many patients are not diagnosed rapidly since there is no specific laboratory diagnostic markers or definitive tests available in the clinic. Efforts have been made to address this issue by unravelling the pathobiology of ME/CFS at both the molecular and clinical levels.

The aetiology of ME/CFS is also poorly understood. The main issue is that ME/CFS is usually diagnosed in the individuals when it is already at its severe stage [5]. About 50-80% of patients show sudden, persistent flu-like symptoms. ME/CFS is commonly occurred following bacterial or viral infections. These infections may render the immune system dysfunctional, initiating ME/CFS that is followed by multi-systemic impairments that may be long-lasting [8, 9]. We speculate that a higher number of COVID-19 patients may increase the reported cases of ME/CFS, as observed following the SARS epidemic [10].

Concerning the pathophysiology of ME/CFS, many studies reported evidence of serotonergic system dysfunction, chronic viral infection, and abnormalities in immune response (*i.e.*, immune activation and chronic inflammation) and cellular bioenergetics [11]. Chronic neuroinflammation near the paraventricular nucleus of the hypothalamus is an important factor in ME/CFS [12]. Evidence from advanced molecular techniques has showed the increased cytokine production, immune activation and inflammation which are associated with deficits in cellular energy metabolism and mitochondrial function. Immune activation can increase flux of tryptophan (TRYP) metabolism *via* the kynurenine pathway (KP) through realising proinflammatory cytokines that induce the activity of indoleamine 2,3-dioxygenase 1 (IDO-1), the primary enzyme in the KP [13]. Up to 90% of TRYP is catabolized through the KP into neuroactive metabolites and nicotinamide adenine dinucleotide (NAD^+^) [14-17]. Metabolites of TRYP catabolism from the KP have been associated with inflammation, immune response, and neurological disorders. It is worth mentioning that any increase in KP activity will shunt away TRYP from serotonin pathways. Downregulation of the serotonin pathway may contribute to emergence of ME/CFS symptoms such as chronic fatigue, depression, sleep problems, and headaches.

The present study is a comprehensive review that i) discusses the possible association of KP with ME/CFS; ii) highlights the role of patient’s gut microbiota in the availability of TRYP; iii) emphasizes the significance of KP metabolites and their relation to disease symptoms, and iv) evaluates evidence for the role of raising levels of NAD^+^ precursors to attenuate disease symptoms. By disseminating this information, this review aims to provide researchers in this field a promising theragnostic concept and bringing us closer to developing a biosignature for ME/CFS.

## 2.Epidemiology of ME/CFS

The exact prevalence of ME/CFS remains controversial; however, a large number of studies reported a significant prevalence of ME/CFS among adults [6, 18-20]. This controversy is due to several factors ranging from differences in the laboratory methodologies and the type of population surveyed for definition and determination of the disease [21-25]. Therefore, a common diagnostic criterion in ME/CFS studies is essential but lacking. Care should be taken to exclude individuals that suffer from known pathological diseases as ME/CFS patients.

**Figure 2.**
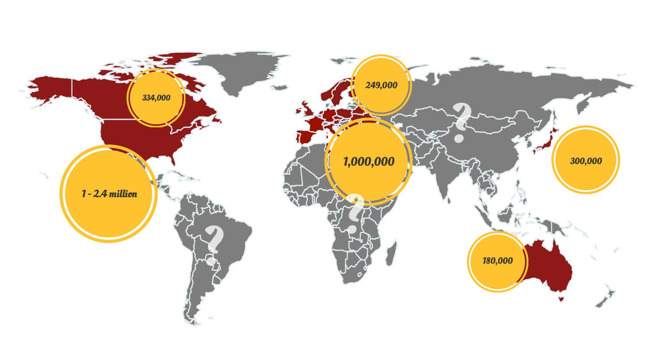
Worldwide population affected by ME/CFS (https://www.emerge.org.au/).

According to the Office of the Privacy Commissioner of Canada (OPC), ~27% of adults in the United Kingdom suffer from chronic fatigue which accounts for the prevalence rate of 13.4% in that population [26, 27]. A study conducted in England revealed an incidence rate of 4.7% (prevalence rate of 0.2%) of CFS among the 143,000 individuals (age, 18-64 years) [28]. Another study in the US estimated that over 6% of people experience significant fatigue that last more than 14 days [29]. Based on the current Centers for Disease Control and Prevention (CDC) statistics, more than one million individuals suffer ME/CFS in the US. According to a four-year study [30] in Wichita, Kansas, the prevalence of CFS was 235 per 100,000 persons, and was four times more common in women than men. Lawrie et al. (1997) [31] reported that the annual incidence and the prevalence of fatigue syndrome in Edinburgh were 370 per 100,000 and 740 per 100,000 individuals, respectively. While definition and classification of the disease vary and can affect the prevalence reports, it has been estimated that about 0.4-1% of Australians suffer ME/CFS [3]. A Norwegian study identified two peaks in age prevalence of ME/CFS. The first one appeared between 10 to 19 years and the other one was observed between 30 to 39 years [32]. Overall, it has been estimated that there are about 17-24 million people with ME/CFS around the world (Fig. 2) with women at four-time higher risk [7, 33].

Approximately, a quarter of patients are totally disabled with an estimated annual cost of $20,000 per individual [34]. Due to complex symptomology, prognosis of patients is challenging. In most of the cases, an improvement of the symptoms is more common than full recovery. [35]. Patients with severe ME/CFS usually die from underlying health complications such as cardiovascular diseases and cancer [36-38].

## 3.Aetiology and pathophysiology of ME/CFS

Several clinical investigations have suggested infections, genetics, physical trauma, stress, and the environment as risk factors for the development of ME/CFS [39-43]. Patients with ME/CFS have several pathophysiological abnormalities that affect various organ systems. However, it is not clear whether these abnormalities occur after ME/CFS or are the initiator of the illness. The possible immune system abnormalities in ME/CFS could include higher production of pro-inflammatory cytokines (*i.e.*,IL-4, IL-5, IL-6, IL-12), immunosuppression due to dysfunctional natural killer cells (NK), decreased function of T cells (e.g. CD8+ and CD4+), and increased production of autoantibodies (*e.g.*, rheumatic factor, anti-thyroid) [25, 44-46]. The severity of ME/CFS may be correlated with poor functioning NK cells. NK cells play key roles in primary recognition and destruction of hijacked cells by viruses. It has been reported in several cases that the susceptibility to viral infections caused by Epstein-Barr virus, cytomegalovirus, and human herpesviruses type 1, 6 and 7 could be increased in ME/CFS due to low-functioning NK cells [39, 47-49]. These infections may, in turn, cause inflammation and/or substantially limit NAD^+^ production. For example, human herpesvirus type 1 infection can trigger degradation of poly(ADP-ribose) glycohydrolase and activation of the NAD-dependent DNA nick sensor poly(ADP-ribose) polymerase (PARP). The latter enzyme consumes NAD^+^ when repairing damages to double strand DNA [50].

Chronic inflammation and oxidative stress in ME/CFS patients may also be triggered by increased production of pro-inflammatory cytokines [51, 52], reduced levels of antioxidant compounds (*e.g.*, coenzyme Q10) and antioxidant enzymes [53], increased oxidative and nitrosative stress (IO&NS) [54, 55], damage to important macromolecules (*e.g.*, DNA, proteins, fatty acids) [56, 57], mitochondrial dysfunction[58], and overactivation of apoptotic pathways [27, 59]. The increase in the levels of pro-inflammatory cytokines during ME/CFS can explain the observed fatigue and flu-like symptoms. However, the most accepted hypothesis for inflammation in ME/CFS is of infectious and particularly viral origin, as a compensatory mechanism to induce anti-viral immune responses and a systemic inflammation [39, 47].

Up to 60% of ME/CFS patients have high titre of autoantibodies [60]. It was indicated that the ME/CFS patients had an IgM-associated immune response against some normally not immunogenic degraded membrane lipids produced by oxidative and nitrosative stress-induced peroxidation and S-farnesyl-l-cysteine [59]. It was suggested that chronic immune system activation and inflammation, probably due to an infection, could cause these autoimmune complications in ME/CFS patients [46, 59]. Increased production of pro-inflammatory cytokines, for example, IL-1 and tumour necrosis factor-α (TNFα), and elevated levels of nuclear factor-κB further deteriorate the autoimmune condition. The number of effector B cells and autoreactive T cells increase in response to abnormal cytokine production [4, 61, 62]. In contrast, the population of NK cells decrease, triggering an imbalance in homeostasis and enhancing the survival of effector T cells [63]. Viral infections, usually observed in ME/CFS patients, can initiate autoimmunity by employing molecular mimicry. Moreover, bacterial translocation which is also seen in these patients can induce autoimmunity and inflammation [64, 65]. Autoimmunity can also be mediated by mitochondrial dysfunction and decreased level of NAD^+^ and ATP that interfere with apoptosis and necrosis [60, 66]. Individuals with ME/CFS have been also identified to possess autoantibodies against neurotransmitters and important components in the central nervous system (CNS) including serotonin, dopamine, gangliosides, muscarinic receptors, and the 5-hydroxytryptamine receptor 1A receptor. These abnormalities coul explain the presence of nervous system-associated symptoms such as chronic fatigue, cognitive impairment, and sleep disturbances [27, 67].

## 4.Tryptophan metabolism and the kynurenine pathway

**Figure 3.**
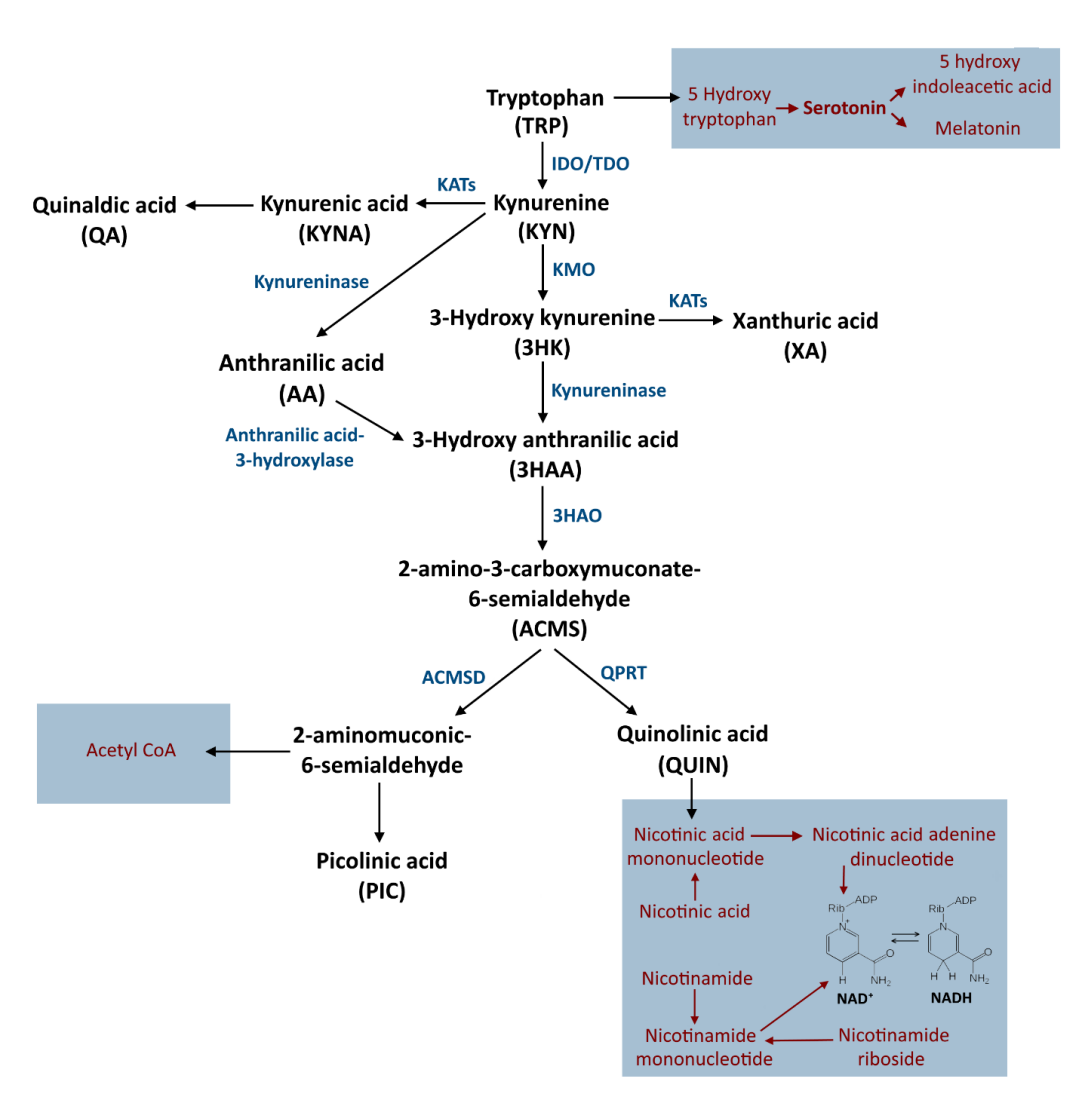
The kynurenine pathway and the NAD+ salvage pathway. IDO: Indoleamine-pyrrole 2,3-dioxygenase; TDO: Tryptophan 2,3-dioxygenase; KATs: Kynurenine aminotransferase; KMO: Kynurenine 3-monooxygenase; 3HAO: 3-hydroxyanthranilate oxidase; QPRT: Quinolinate phosphoribosyl transferase; ACMSD: ACMS decarboxylase.

TRYP is an essential amino acid which is a precursor for various bioactive molecules, the most noteworthy of which is serotonin. However, a small proportion of TRYP is converted into serotonin and more than 90% of that is metabolised to kynurenine (KYN), several downstream neuroactive metabolites, and *de novo* synthesis of NAD^+^. The KP is strongly induced by pro-inflammatory cytokines such as INFγ [14, 15]. Two important enzymes including tryptophan dioxygenase (TDO) and IDO-1 catalyse the first rate-limiting step of the KP that is the conversion of TRYP to KYN. TDO is mainly expressed in the liver while IDO-1 is present in many cells such as macrophages and brain cells [14, 16, 68]. Under normal physiological conditions, KYN is mainly converted to 3-hydroxykynurenine (3-HK) by the catalytic activity of the enzyme kynurenine monooxygenase (KMO). In the next steps, 3-HK is metabolized to 3-hydroxyanthranilic acid (3HAA), quinolinic acid (QUIN), and finally NAD^+^. The remaining KYN is also converted into kynurenic acid (KYNA) by kynurenine aminotransferase (KAT) isozymes. KYNA is known as a neuroprotective metabolite antagonising the excitotoxic potential of QUIN (Fig. 3).

### 4.1 Alteration of tryptophan metabolism in ME/CFS

#### 4.1.1 Depression and mood disorders

Several studies have provided evidence that overactivation of IDO-1 and subsequent TRYP depletion may be associated with depression and other mood disorders [69-72]. Mood disorders are prevalent complications in ME/CFS patients [59, 73, 74]. IDO-1 is extensively expressed in different human tissues including the kidney, brain, lungs, dendritic cells, and macrophages. It is induced by IFN-γ, TNFα, IL-1β, amyloid β, and lipopolysaccharide [14, 15]. It is well-documented that mood disorders are linked with cell-mediated immune system activation, which is accompanied by increased inflammation, production of IFN-γ, IL-1β, and TNFα [69, 75]. Theses pro-inflammatory cytokines can strongly activate the KP. Overactivation of the KP diverts TRYP from the serotonin biosynthesis pathway, thus depleting serotonin levels while stimulating overproduction of KP metabolites. It should be noted that KYN has depressogenic properties, whereas KYNA is a neuroprotective metabolite. Therefore, the ratio of KYN to KYNA is a significant indicator of KAT activity and neurotoxic potential. Accordingly, Maes et al. (2011) [76] reported that depressive symptoms, particularly somatization, is directly associated with increased levels of serum KP metabolites as specified by the KYN:KYNA and KYN:TRYP ratios, and negatively relates to levels of serum TRYP in serum [76].

The reduced conversion of KYN into KYNA can be due to glial depressing factors that downregulate the activity of KAT-I [77, 78]. This phenomenon can lead to excessive levels of KYN and QUIN. Alternatively, the increase in KYN:KYNA ratio could be justified by the effect of microglia on astrocytes during inflammation. Chronic inflammation, which is widely observed in ME/CFS patients, activates both microglia and astrocytes [79]. These cells are considered as the major sites of TRYP catabolism [80]. Astrocytes are the main KYNA-producing cells whereas microglia are the main QUIN producers [81, 82]. Microglia activation induces KP in the brain and produces pro-inflammatory mediators [83]. The overproduction of QUIN by activated microglia, in turn, decreases KYNA levels by supressing astrocytes. High microglial populations and low astrocyte titre was reported in depressive individuals [84-86]. It has been suggested that microglia are activated in large parts of the brains of ME/CFS patients. Accordingly, Nakatomi et al (2014) [87] reported an increase in the binding potential values of ^11^C-(R)-PK11195, a ligand for a translocator protein that is expressed by activated microglia, in patients with ME/CFS compared to the healthy controls.

Microglial activation can also trigger upregulation of serotonin transporter (5-HTT) in astrocytes *via*releasing IL-1β, decreasing the levels of extracellular serotonin leading to depression. The interaction between IL-1β released from microglia and astrocytic 5-HTT is well-known, proved by employing IL-1 receptor antagonists. IL-1β can directly induce the upregulation of 5-HTT in primary cultured rat astrocytes [88]. This pro-inflammatory cytokine has also been detected in significantly high concentrations in ME/CFS patients, which is produced by activated microglia [89].

#### 4.1.2QUIN production and NAD^+^ metabolism in ME/CFS

Besides astrocyte suppression, overproduction of QUIN induces oxidative stress, neuroinflammation, mitochondrial dysfunction and cell death. Activated microglia and infiltrating macrophages are the main source of QUIN production in the brain. Like glutamate, QUIN is an agonist of the N-methyl-D-aspartate receptor (NMDAR); therefore, its accumulation can induce excitotoxicity in neurons and astrocytes. A potent mechanism of QUIN neurotoxicity is through lipid peroxidation. More specifically, QUIN can form a complex with iron that mediates the formation of reactive oxygen species (ROS) and reactive nitrogen species (RNS) and cause oxidative damage to lipids, proteins, and nucleic acids. Many studies have reported increased levels of oxidative stress in ME/CFS patients, promoting lipid peroxidation and protein carbonyl formation [43, 90, 91]. Oxidative and nitrosative stress induce damage to endogenous epitopes (*e.g.*, residue components of lipid peroxidation), and trigger secondary IgM and IgG mediated autoimmune responses against neoepitopes [92, 93]. QUIN could also increase the activity of inducible nitric oxide synthase (iNOS) and nitric oxide (NO) production, which contributes to the activation of immune cells in the brain. ME/CFS patients have significantly higher iNOS activity, compared to healthy people [92, 94]. Consequently, increased NO production is also expected in these patients [95].

Alternatively, excessive QUIN production in ME/CFS patients could negatively impacted NAD^+^ metabolism. TRYP degradation represents the *de novo* route of NAD^+^ production, and *de novo* NAD^+^ synthesis increases in chronic inflammatory conditions. NAD^+^ levels increase concomitant with increases in QUIN up to levels where saturation of the enzyme quinolinic acid phosphoribosyltransferase (QPRT) occurs. The cytotoxic effects of QUIN have been reported at higher levels. NAD^+^ plays an important role in the cellular respiratory chain and some other cellular processes (*e.g.*, calcium homeostasis, apoptosis, ageing, DNA repair, immunogenicity, transcriptional regulation) [96-99]. Several studies have demonstrated that QUIN-induced oxidative stress could overactivate poly (ADP-ribose) polymerase (PARP), an enzyme that repairs damaged DNA following exposure to oxidative insult [98, 100, 101]. PARP overactivation depletes NAD^+^ and ATP, and significantly contributes to mitochondrial dysfunction, cell energy loss, and overproduction of ROS and RNS *e.g*., NO and superoxide, which are observed in ME/CFS.

Studies using animal models that reflect some of the clinical features of ME/CFS have indicated a strong association between decreased expression of mitochondrial complexes, changes in mitochondrial morphology, and ultimately mitochondrial dysfunction with fatigue-like performance, high pain sensitivity, and depression [102-104]. A recent Sequential Window Acquisition of All Theoretical Mass Spectra (SWATH-MS) analysis of ME/CFS peripheral blood mononuclear cell proteome identified a distinct decline in ATP and energy production in ME/CFS and an upregulation of Complex V in the mitochondrial respiratory chain, suggestive of increased ROS production [105]. Deficiencies in the natural coenzyme Q10 (CoQ10) and NADH, the reduced form of NAD^+^ have been reported in patients with ME/CFS [106]. Optimal levels of CoQ10 and NADH are required for mitochondrial oxidative phosphorylation and ATP production.

#### 4.1.3Gut microbiota and ME/CFS

The human microbiota is a dynamic community comprising more than 10,000 different microbes including bacteria, archaea, viruses, fungi, and protozoa. Naturally, the microbiota is commensal or symbiotic flora which have been co-evolved with their host and play important roles in various physiological process such as immune responses and nutrition [14, 107]. More specifically, the human gastrointestinal (GI) microbiota contains a complex microbial ecosystem which strongly maintain homeostasis of the GI tract. It has been suggested that dysbiosis of the GI microbial population could be associated with ME/CFS [108-110]. Giloteaux et al [109] reported that GI microbial diversity in ME/CFS patients was lower, compared to healthy controls. The study also found higher levels of some inflammatory biomarkers including bacterial LPS, CD14, and LPS-binding protein in the blood samples of ME/CFS patients. For example, IL-1β, IL-6, IL-8, and TNFα are some of the pro-inflammatory cytokines that were increased during ME/CF [109]. Another study reported that up to 92% of ME/CFS patients suffer from Irritable Bowel Syndrome (IBS) [111].

TRYP can be metabolized either directly or indirectly by gut microbiota to several compounds such as serotonin, kYN, indolyl compounds, and tryptamine, which play important roles in gut-brain axis communication [14, 112, 113]. More specifically, *in silico* analyses have indicated that some GI bacterial species (*e.g.* phyla Bacteroides, Firmicutes, Fusobacteria, Actinobacteria, Proteobacteria) can catabolize TRYP in the GI system and produce neuroactive metabolites including KYN, KYNA, and QUIN [14, 114]. Compared to other GI bacteria, genera *Burkholderia*, *Ralstonia*, *Klebsiella*, and *Citrobacter*have a higher potential to catabolize TRYP to neuroactive compounds [114]. Microbial indole and indole-derived compounds (*e.g.*, indole-3-aldehyde, indole-3-acetic acid, indole-3-propionic acid, indole-3-acetaldehyde) could regulate GI homeostasis as well as IDO-1 expression. These indolyl compounds play important roles in GI as signalling molecules, transferring information among the GI, innate and adaptive immune system, various immune cells such as NK cells, T-cells, and macrophages [15, 115] through binding to the aryl hydrocarbon receptor (AhR) [14, 15]. Indoles and their derivatives are known as natural ligands of AhR, a ligand-activated transcription factor which mediates the communication between the host and microbiota [116]. The ligand-AhR complex is translocated into the nucleus and bound to AhR nuclear translocator (ARNT) protein to form a heterodimer structure inducing specific genes containing a sequence named aryl hydrocarbon response elements (AhREs) [116]. The AhR-ARNT complex can also regulate the expression of IL-6 in macrophages. IL-6 subsequently induces the expression of IDO-1 through JAK/STAT signalling [117], increasing the levels of TRYP metabolites and enhancing TRYP depletion [14]. Increasing the level of IL-6 in ME/CFS patients has been reported in several studies [89, 116, 118] and plays a role in development of ME/CFS symptoms. Therefore, the gut microbiota can potentially affect the level of IL-6 and inflammation in ME/CFS patients through consuming TRYP.

As mentioned earlier, it has been reported that intestinal microbial biodiversity decreases in ME/CFS patients [108, 109, 119, 120]. Decreased gut microbial diversity could affect the levels of circulating TRYP as well as KYN metabolism in the peripheral and central nervous systems [14, 116]. Frémont et al [116] reported significant differences in gut microbial composition in ME/CFS patients and healthy individuals, with increased number of genus *Alistipes* within Bacteroidetes phylum. Another study conducted by Lupo et al [121] reported increased abundance in genera Bacteroides and a reduction in Firmicutes (except *Lactonifactor*) population in ME/CFS patients. Several species of genus Bacteroides (*e.g. B. thetaiotaomicron, B. ovatus, B. eggerthii, B. fragilis*) have been recognized to produce indole and its derivatives from TRYP catabolism in the gut [116]. It could be hypothesized that increased abundance of genus Bacteroides in the GI of ME/CFS patients may enhance TRYP metabolism and indole production leading to TRYP depletion. The reduction in Firmicutes population [108] can decrease the production of short chain fatty acids (SCFAs) in ME/CFS patients’ guts. Butyrate, a SCFA with anti-inflammatory activity, decreases IDO-1 transcription through inhibition of histone deacetylase and decreasing the expression of signal transducer and activator of transcription 1 (STAT1) leading to inhibition of IFN-γ dependent STAT1 phosphorylation and finally decreasing the STAT1-driven transcriptional activity of IDO-1 [122]. Consequently, IDO-1 could be more active in ME/CFS patients, compared to the healthy individuals, due to lack of butyrate-producing bacteria.

## 5. NAD^+^ as a therapeutic target in ME/CFS

The activity of pro-inflammatory cytokines is the driving force for increased TRYP catabolism through the KP. Therefore, strategies aimed at suppressing the activity of pro-inflammatory cytokines, for example, synthesis of IFN-γ in the peripheral IL-6 in the CNS can counteract TRYP depletion. However, TRYP depletion also represents an important mechanism to starve pathogens and tumour cells and facilitate immune tolerance [123]. Moreover, therapeutic strategies aimed at inhibiting the KP can have a dramatic effect on *de novo* NAD^+^ synthesis [124].

Apart from the KP, NAD^+^ can also be produced from the salvage pathway using the precursors nicotinic acid (NA), nicotinamide (NAM), nicotinamide mononucleotide (NMN) and nicotinamide riboside (NR) [125]. However, it should be noted that NA therapy induces some unwanted negative adverse effects including significant skin flushing in most individuals below therapeutic doses, which limits its widespread clinical use [125]. NAM is the primary form of vitamin B3 found in meat and is synthesized as a by-product of enzymatic degradation of NAD^+^ by PARPs, Sirtuins and NAD^+^ glycohydrolases (*e.g* CD38) [125]. NAM aids recovery from depression and mood disorders by modulating monoamine-neurotransmitter synthesis and degradation, potent antioxidant effects and increasing intracellular NAD^+^ levels [126]. Although supplementation with NAM raises NAD^+^ without causing flushing, it is unlikely to be an ideal NAD^+^ supplement due to its enzyme inhibiting (e.g., PARPs, sirtuins, CD38), methyl depleting and hepatotoxic potential [125].

NMN can be produced endogenously from NR [125]. Animal studies have shown that NMN can ameliorate degeneration and improved age-related cognitive decline [127-131]. However, the intracellular uptake of NMN is unclear. Recently, one study reported that NMN is dephosphorylated to NR and then internalization by the solute carrier family 12 member 8 (Slc12a8) [132]. However, another report did not find enough evidence to support Slc12a8 as the ‘reclusive’ NMN transporter [133].

NR is a precursor that can be naturally obtained by regular consumption of cow milk [125]. NR is converted to NAD^+^ via the catalytic activity of NRK1 and NRK2. There also exists an NR kinase independent pathway which converts NR to NAM which is then salvaged back to NAD^+^ [125]. NR has been shown to prevent cognitive decline and ameliorate brain attenuate brain degeneration [126], and correct social deficits and anxiety disorders in animal models [134]. This suggests that NR may have beneficial effects in ME/CFS. Oral uptake of NR has been reported to increase NAD^+^ concentrations in whole blood and tissue in humans. NR is safe and more orally bioavailable than other NAD^+^ precursors [125]. Unlike other niacin supplements, NR does not cause flushing. However, there are no clinical trials using NAD^+^ precursors, particularly NMN and NR in ME/CFS. Further clinical evidence is necessary for the use of NMN and NR as NAD^+^ supplements for managing and preventing ME/CFS.

Mitochondrial dysfunction, which limits NAD^+^ and ATP levels, is the central agent for energy production in patients with ME/CFS. Supplementation with CoQ10 (200 mg/day) and NADH (20 mg/day) can enhance cellular bioenergetics and reduce fatigue and lipoperoxides, and improve biochemical parameters (e.g. CoQ10, ATP, citrate synthase and NAD^+^:NADH redox ratio, in humans) [135]. One study showed a significant reduction in anxiety and maximum heart rate in a Spanish CFS cohort [136]. Another study demonstrated a significant reduction in symptoms following treatment with NADH (10 mg/day), compared to placebo [137]. Oral supplementation with NADH was more effective in terms of symptom reduction than conventional nutritional supplements and psychotherapy over a 24-month period [138]. Oral NADH has been shown to be safe and well tolerated in humans [135-138]. However, results are conflicting for CoQ10. One study showed that supplementation with CoQ10 (50 mg/day) and NADH (5 mg/day) significantly reduced maximal heart rate during cycle ergometer tests. Fatigue perception was also reduced, although no improvements were reported for pain and sleep [139]. However, larger controlled trials are needed to confirm these findings.

## 6. Conlusions

ME/CFS is a poorly understood disorder with unknown aetiology and no established diagnostic criteria. During ME/CFS, NAD^+^ level significantly decrease, leaving the patient with severe fatigue. This drop can be largely explained by 1) the overactivation of IDO-1 shunts the available TRYP into KP, increasing KP metabolites, depleting serotonin, and causing mood disorders and other related neurological symptoms; 2) QUIN, at pathophysiological concentrations can trigger oxidative stress and apoptosis *via*excitotoxicity in different cells including neurons and astrocytes. The lower population of astrocytes, in turn, is translated into lower KYNA production, further enhancing neurological symptoms; 3) The available NAD^+^ and ATP are depleted due to overactivation of PARP, following QUIN-induced oxidative damages to DNA. This phenomenon significantly contributes to cell energy loss and leads to mitochondria dysfunctional; 4) Mitochondrial dysfunctional further promotes the production of ROS and RNS leading to oxidative stress and reduced function; and 5) The accumulation of QUIN can have positive feedback control on all the mechanisms explained above by triggering chronic inflammation. This chronic inflammation is due to QUIN-induced damage to macromolecules, ROS and RNS-induced damage to endogenous epitopes, and secondary IgM and IgG mediated autoimmune responses against neoepitopes. Alteration to the gut microbiota may further contribute to ME/CFS through increased production of indole and its derivatives and modified availability of circulatory TRYP. Reduction of microbial biodiversity in GI of ME/CFS patients may increase inflammation and IDO-1 activity leading to increased TRYP catabolism and depletion.

As well, raising intracellular NAD^+^ levels can improve the quality of life by improving neurological function, promoting energy production, and lowering fatigue. NAD^+^ precursors such as NR and NMN have been suggested as potential treatments to correct anxiety disorders, fatigue, and social deficits in animal models. However, there are no clinical trials using NAD^+^ precursors to alleviate the clinical features of ME/CFS and further clinical assessment are needed for the use of these precursors for increasing the level of NAD^+^ in ME/CFS patients. Supplements using NADH and CoQ_10_ are safe and well-tolerated and may be added to conventional CFS therapy subject to recommendation from a general practitioner.
